# Cardiac disease discrimination from 3D-convolutional kinematic patterns on cine-MRI sequences

**DOI:** 10.7705/biomedica.7115

**Published:** 2024-05-31

**Authors:** Alejandra Moreno, Lola Xiomara Bautista, Fabio Martínez

**Affiliations:** 1 Biomedical Imaging, Vision, and Learning Laboratory (BIVL2ab), Universidad Industrial de Santander, Bucaramanga, Colombia Universidad Industrial de Santander Universidad Industrial de Santander Bucaramanga Colombia

**Keywords:** Heart diseases, diagnostic imaging, magnetic resonance spectroscopy., cardiopatías, diagnóstico por imagen, espectroscopia de resonancia magnética.

## Abstract

**Introduction.:**

Cine-MRI (cine-magnetic resonance imaging) sequences are a key diagnostic tool to visualize anatomical information, allowing experts to localize and determine suspicious pathologies. Nonetheless, such analysis remains subjective and prone to diagnosis errors.

**Objective.:**

To develop a binary and multi-class classification considering various cardiac conditions using a spatiotemporal model that highlights kinematic movements to characterize each disease.

**Materials and methods.:**

This research focuses on a 3D convolutional representation to characterize cardiac kinematic patterns during the cardiac cycle, which may be associated with pathologies. The kinematic maps are obtained from the apparent velocity maps computed from a dense optical flow strategy. Then, a 3D convolutional scheme learns to differentiate pathologies from kinematic maps.

**Results.:**

The proposed strategy was validated with respect to the capability to discriminate among myocardial infarction, dilated cardiomyopathy, hypertrophic cardiomyopathy, abnormal right ventricle, and normal cardiac sequences. The proposed method achieves an average accuracy of 78.00% and a F_1_ score of 75.55%. Likewise, the approach achieved 92.31% accuracy for binary classification between pathologies and control cases.

**Conclusion.:**

The proposed method can support the identification of kinematically abnormal patterns associated with a pathological condition. The resultant descriptor, learned from the 3D convolutional net, preserves detailed spatiotemporal correlations and could emerge as possible digital biomarkers of cardiac diseases.

## Resumen

**Introducción.:**

Las secuencias del cine-resonancia magnética (*cine-MRI, cine magnetic resonance imaging*) son una herramienta diagnóstica clave para visualizar la información anatómica que les permite a los expertos localizar y determinar aquellas anomalías que resulten sospechosas. No obstante, este análisis sigue siendo subjetivo y propenso a errores de diagnóstico.

**Objetivo.:**

Desarrollar una clasificación binaria y multiclase, considerando diferentes condiciones cardiacas, mediante un modelo espaciotemporal que permita resaltar los movimientos cinéticos para caracterizar cada enfermedad.

**Materiales y métodos.:**

Este estudio se centra en el uso de una representación de convolución 3D para caracterizar los patrones cinéticos durante el ciclo cardiaco que puedan estar asociados con enfermedades. Para ello, se obtienen mapas cinéticos a partir de mapas de velocidad aparente, calculados mediante una estrategia de flujo óptico denso. A continuación, un esquema de convolución 3D "aprende" a diferenciar patologías a partir de mapas cinemáticos.

**Resultados.:**

La estrategia propuesta se validó según la capacidad de discriminar entre infarto de miocardio, miocardiopatía dilatada, miocardiopatía hipertrófica, ventrículo derecho anormal y un examen normal. El método propuesto alcanza una precisión media del 78,0 % y una puntuación F_1_ *score* del 75,55 %. Asimismo, el enfoque alcanzó el 92,31 % de precisión para la clasificación binaria entre enfermedades y casos de control.

**Conclusiones.:**

El método propuesto es capaz de apoyar la identificación de patrones cinéticos anormales asociados con una condición patológica. El descriptor resultante, aprendido de la red de convolución 3D, conserva correlaciones espaciotemporales detalladas y podría surgir como posible biomarcador digital de enfermedades cardiacas.

Associated cardiac pathologies represent the main cause of death worldwide, representing around 30% of the total deaths ^1^. The movement and kinematic components of cardiac structures represent a key biomarker of heart disorders. Magnetic resonance imaging (MRI) has become the primary clinical diagnostic technique for quantifying, inspecting, and analyzing the heart. The ejection fraction can be calculated from the MRI modality to discriminate among several cardiac conditions. However, the estimation of such measurements is based on manual delineation, which can be subject to errors. In addition, cardiac measurements may be insufficient to characterize and differentiate the diverse cardiac behaviors, often complex among different cardiac diseases.

Computational methods have allowed for modeling and quantifying the motion and shape of cardiac features, supporting tasks related to segmentation ^2^^-^^5^, motion analysis, and classification of cardiovascular diseases. Regarding segmentation, the approaches have used atlas templates ^2^^,^^6^, encoder-decoder architectures ^7^^-^^9^, and even deep representations dedicated to localizing regions ^3^.

Likewise, Qin *et al*. used a motion-seg net to simultaneously obtain motion and shape estimations under an unsupervised scheme ^10^. Additionally, semi-supervised learning was introduced to propagate cardiac disease labels, using as a backbone a U-net that codifies the shape and motion features ^11^. Punithakumar *et al*. calculated diverse statistics related to velocities and ventricle distances to classify pathologies, such as infarcts, dilated heart disease, and other cardiovascular diseases ^12^. Also, Zheng *et al*. performed an unsupervised cardiac image representation learned from a multi-scale deep network that achieved a direct volume estimation ^13^.

The present work introduces a deep volumetric representation that fully characterizes cardiac motion patterns, allowing motion embedding descriptors that classify diverse cardiac diseases. From deep cardiac representation, the high-level net embedding vectors are obtained as hidden kinematic cardiac descriptors used to classify and discriminate among several cardiac pathologies.

In the next sections, we will describe the methodological approximations and the validation over a public dataset.

## Materials and methods

This work introduces a 3D convolutional representation to encode cardiac kinematic maps as embedding descriptors that can classify cardiac condition set. From velocity fields, cardiac kinematic maps are calculated to locally represent patterns such as normal and tangential acceleration, divergence, and vorticity. These enriched and dense motion primitives are convolved several times to obtain a hierarchical deep representation of 2D+t spatiotemporal patterns through the cardiac cycle along the short axis.

**Figure 1.**
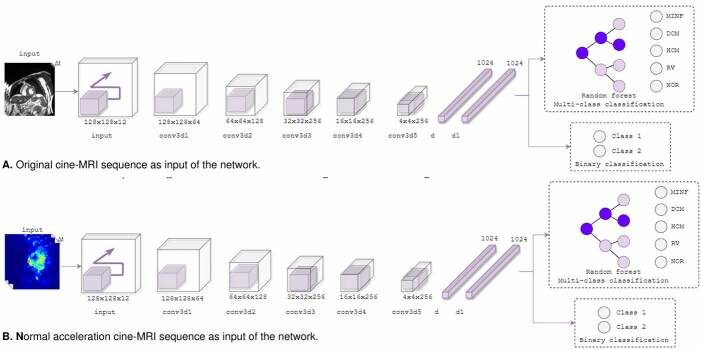
Pipeline of the proposed representation to classify heart conditions from cine-MRI temporal sequences (bottom-up scheme) or using kinematic maps as input on deep representation.

The main hypothesis underlying this work is the capability of spatiotemporal motion patterns to represent cardiac conditions. In consequence, the architecture can receive (2D +) feature maps of the whole cardiac cycle, generating a hidden deep and latent representation that discriminates among different cardiac diseases. The general pipeline of the proposed approach is shown in figure 1.

### Kinematic cardiovascular maps

To characterize the motion patterns in the cardiac cycle, it is necessary to build a set of kinematic maps that recover motion features from a dense optical flow strategy. The displacement vector field, computed among consecutive frames, is related to the apparent velocity of the cardiac cycle. Here, the displacement vector field was selected as an approximation of the optical flow that recovers large displacements, as well as a deformation representation that lies in a constraint across nearby regions.

For each pair of consecutive frames I(x)_t_,I(x)_t+1_, a dense motion field was computed ⱱ := (u, v)^t^. From this motion field, we computed * = (x,y)^T^, a respective displacement vector (u, v) for each pixel. Hence, the dense motion field was obtained as a typical minimization of appearance (llI_t_ - I_t+1_ll^2^) and gradient (ll▽_f_ - ▽_t+1_ll^2^), with a function that matches non-local points (SIFT points) where the fiow region is coherent ^14^.

The kinematic maps are then derived from this optical flow field and are represented as k = [k_1,_ k_2_,...k_i_]. In this case, k represents a motion feature map, while i is the Index of each calculated kinematic (velocities, accelerations, divergences, or vorticities). It is important to note that these feature maps can be used as isolated observations or even integrated to enrich cardiac disease representation from motion patterns.

Initially, we considered two acceleration types: The first one Is the normal acceleration, representing the direction change of the velocity considering as reference the local center of rotation of the analyzed point 
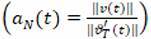
 ; the second was tangential acceleration, introduced to approximate heart deformation during the cardiac cycle as: 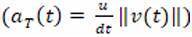 .

We also included divergence and vorticity. Divergence measures the motion density of the input compared to the output and mathematically is formulated as: 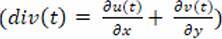 ; vorticity measures the cardiac rotational motion during contraction (ED) and relaxation phases (ES) and is defined as: 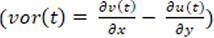


### A deep 3D convolution architecture

A key issue in cardiac conditions analysis is the modeling and quantification of spatial and temporal patterns, allowing the identification of correlations between sequence observations and specific pathologies. In this study, we implemented a robust 3D convolutional representation, which captures spatiotemporal patterns at different processing levels through a hierarchical convolutional configuration ^15^. Hypothetically, we assumed that a cine-MRI observation can be fully expressed by spatiotemporal patterns. These patterns are incorporated into a deep representation adjusted through a conditional discrimination rule. Interestingly, the proposed architecture can receive kinematic maps to code more complex relationships related to cardiac conditions.

**Figure 2.**
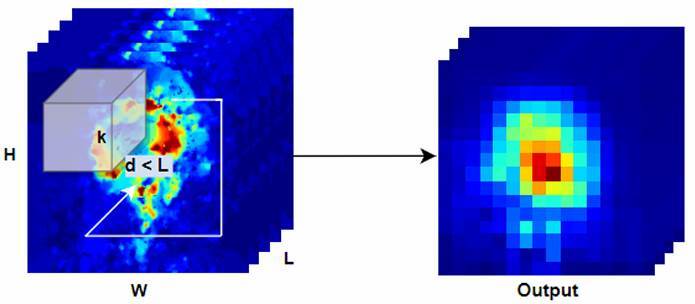
3D convolution representation. An input image of size L x H x W performing a convolution with a kernel *t* ^*^ *y* ^*^ *x*.

A sequence of images ɸ of dimension (*L x H x W*) stands for either a cine- MRI sequence, the corresponding kinematics representation (velocities, accelerations, vorticity, or divergence), or even a concatenation of multiple kinematics. In this case, l denotes the temporal frame number of the cardiac cycle, and (*H x W*)denotes the spatial frame dimensions. This sequence is then used as input in the convolutional representation and operated at different 3D-convolutional layers. In such case, k exemplifies the -dimensional convolution kernel, where the z dimension convolves over the temporal axis and the (*v, w*) dimensions over the spatial axes. At each processing step, we calculated a representation volume o’ indicating a bank of spatiotemporal feature maps, capturing a more accurate characterization of motion throughout the cycle (figure 2).

The total feature volume represents the union of each map b for the specified number D^1^ of kernels. The respective generalization at different layers provides a multi-scale motion representation providing a signature for each cardiovascular disease.

### A cardiac embedding representation

The proposed convolutional representation can predict cardiac conditions under an end-to-end scheme, embedding a probability prediction output within the final layer according to training cardiac classes. Furthermore, the final layers of the architecture model are a set of hidden and complex relationships of kinematic inputs, representing a descriptor of a particular disease.

In this work, we explored the embedding space resulting from these descriptors and measured the capability to discriminate among pathologies. Specifically, we extracted the embedded vectors from the last dense connected layer. For a particular dataset, the inputs are then mapped to a trained net, and the compact vectors are recovered with the label corresponding to the disease y.

The training sample set was used to create a random forest, defined as 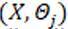 , where 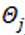 represents each decision tree of independent and identically distributed random variables formed by a uniform random selection of characteristics. A particular threshold (π_i_) is learned for each kinematic (k), which creates a node in the tree 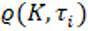 and builds a new partition in the feature space.

The group of trees gives an independent vote for predicting pathology draw a discrete partition over topological space to define the boundaries of cardiac classes and obtain an automatic classification.

### Experimental setup

This section details the data and procedures of the proposed approach to validate the method and its performance according to the classification task. The next subsection describes each of the components in the experimental setup.

*ACDC database:* This method was tuned and validated with a public dataset named Automated Cardiac Diagnosis Challenge (ACDC) ^16^. The dataset consisted of a cine-MRI image set from patients diagnosed with cardiovascular diseases and a control population. Four pathologies are characterized by ejection fraction and other morphological features. The myocardial infarction is defined by multiple myocardial segments with an abnormal contraction and a left ventricular ejection fraction of less than 40%. The dilated cardiomyopathy is depicted as having a left ventricular ejection fraction of less than 40% and a diastolic left ventricular volume greater than 100 ml/m^2^. Several myocardial segments with a thickness greater than 15 mm in diastole, a left ventricular cardiac mass greater than 110 g/m^2^, and a normal ejection fraction constitute indicators of hypertrophic cardiomyopathy. On the other hand, when a patient has a right ventricular cavity volume greater than 110 ml/m^2^ and a right ventricle ejection fraction lower than 40%, it indicates an abnormal right ventricle cardiac condition. This dataset also included patients labeled with a normal cardiac condition.

For whole image sequences recorded in the dataset, the heart position is mainly on the basal and the mid-cavity. Each patient has a mean of 9 slices (from apical to basal), varying from 13 to 56 temporal frames across the cardiac cycle. The study includes 100 patients diagnosed with one of the described pathologies (20 patients for each cardiac condition) and an estimated number of slices of 1,300. From a data analysis study, the cardiac cycle for each volume was set to 13 temporal frames. Volumes with larger cardiac cycles were sub-sampled, ensuring the coverage of the end-diastole and end-systole.

Implementation details: The configuration of the proposed 3D convolutional architecture is summarized in table 1. All the convolutions have the same size (2X2X2) except for the initial (1X2X2). For the introduced method, we configured two different strategies of classification, described as follows:

**Table 1.** Parameters of the 3D deep convolutional architecture

Layers	Output shape	Parameters	Activation
*Input*	(12, 128, 128, 1)	-	ReLU
*Conv3D*	(12, 128, 128, 64)	5,842	ReLU
*Conv3D*_*1*_	(6, 64, 64, 128)	221,312	ReLU
*Conv3D*_*2*_	(3, 32, 32, 256)	884,992	ReLU
*Conv3D*_*3*_	(2, 16, 16, 256)	1,769,728	ReLU
*Conv3D*_*4*_	(1,4, 4, 256)	1,769,728	ReLU
*Dense*	1,024	1,049,600	ReLU
*Dense*_*1*_	1,024	1,049,600	ReLU
*Dense*_*2*_	2	2,050	ReLU

- End-to-end training: The 3D convolutional approach was constructed with a softmax layer to classify the cardiac pathologies. The net was trained with a batch of one, a 0.001 learning rate, and an Adam optimization. The proposed net was also adjusted with a dropout of 0.4 and batch normalization to prevent over-fitting and regularize the loss. We used 20 epochs and followed a binary classification rule during each run.
- Random forest classifier: We used the activations from the embedding layer of the learned net. It was expected that such embedding encoded learning kinematic features and allowed discrimination among cardiac conditions. The embedding descriptor was taken from the layer Dense, serving as input to a random forest. In such cases, all the kinematics were trained independently for each discrimination rule between two cardiac conditions. Hence, each image was mapped to each architecture, obtaining the respective embedding vector corresponding to the last layer. The corresponding embeddings are concatenated, representing the new cardiac descriptor of each input sample. A fine- tuning was performed with the following parameters:

1. A maximum tree of 100 and a maximum depth of 60;
2. Each tree was encoded in a binary classification for experiments to discriminate between pairs of classes; and
3. Each tree encoded a multi-class classification between normal cardiac sequences versus any cardiac pathology.

### Statistical validation

The proposed strategy was validated according to the “leave-one-patient- out” scheme, adapted from the classical leave-one-out cross-validation. In this case, one patient (with 13 slices) was left out for testing purposes while the rest of the patients (39 subjects accounting for 507 slices for each binary classification) were used for tuning the model until all patients were validated. For end-to-end experiments, a convolutional net was trained at each fold and later validated with samples of a particular patient. The averaged results corresponded to the reported performance on classification.

When the validation scheme was finished, the model retrieved a prediction for each patient, helping to account for each metric classification, such as accuracy, precision, sensitivity, or F, score. Worth noticing is that each iteration had no overlap between patients since only one cardiac cycle was used per patient. The input samples have a dimension of 12,128,128,1. For experiments with multiple kinematics, the multidimensional input was set as 12,128,128, 3. Each dimension corresponds to the cardiac cycle, height, width, and the concatenation between kinematics. The best multi-kinematic representation was considered to obtain descriptor vectors to perform experiments from embedding representation.

## Results

### Classification from an end-to-end scheme

**Figure 3.**
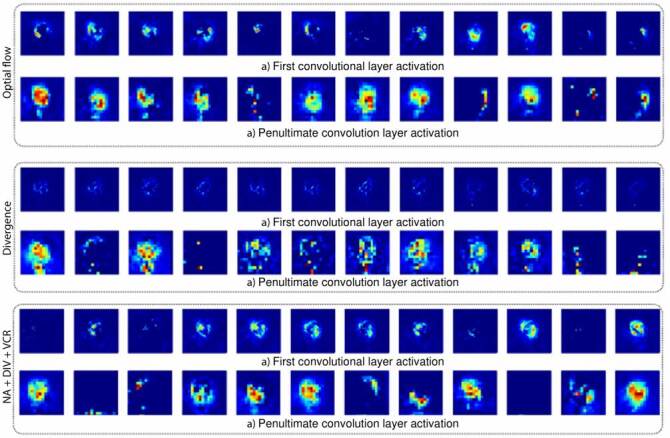
Feature map representation in the convolutional layers obtained from the first and penultimate layers. These primitives are the optical flow, divergence, and a concatenation between normal acceleration, divergence, and vorticity. The illustrated sample corresponds to cine-MRI labeled as a myocardial infarction.

Figure 3 illustrates corresponding heart-kinematic activations from two layers of the proposed architecture. As expected, these maps enhance spatial relationships that eventually may correspond to patterns associated with a specific disease. The illustrated sample corresponds to cine-MRI labeled as a myocardial infarction condition. As inputs, the optical flow channels and the divergence were included independently.

Also, we mapped the normal acceleration, divergence, and vorticity combinations onto the trained architecture to obtain deep hierarchical activations. For each illustrated input, these activations achieved consistent localization that stands out in particular kinematic behaviors at ventricles during a cardiac cycle. These activations hierarchically code cardiac descriptors for an automatic classification to be supported and can be implemented as observational maps to develop further analysis during diagnosis and clinical routine. For three input configurations, the activations of the first layer highlighted local cardiac patterns, while the (L-1) layer focused on the coarse characterization of heart regions. These maps involved temporal correlations allowing an enriched heart description during the cardiac cycle.

Table 2 summarizes the classification performance of the proposed approach using independent kinematic cine-MRI features. The accuracy and the F, score were the metrics selected to analyze globally the performance of the proposed descriptor. As observed, each independent kinematic can discriminate between two cardiac conditions, being potential descriptors to support disease diagnosis. On average, the velocity field patterns (accuracy of 75.83% and f, score of 71.50%) and the divergence (accuracy of 75.23% and f, score of 72.34%) achieved better discrimination for all the experiments.

**Table 2.** Accuracy and F_1_ score obtained using the Automated Cardiac Diagnosis Challenge dataset in the deep learning strategy.

Cardiac diseases	a_T_ *(t)*		a_N_ *(t)*		Divergency		Vorticity		Optical flow		Cine-MRI	
	ACC	F1	ACC	F1	ACC	F1	ACC	F1	ACC	F1	ACC	F1
MINF vs. DCM	82.50	83.72	80.00	80.00	70.83	77.57	85.00	84.21	89.20	85.00	81.20	72.50
MINF vs. HCM	65.20	61.53	70.00	70.00	70.20	77.57	62.50	61.53	73.09	65.00	59.09	57.50
MINF vs. RV	80.00	77.77	80.00	78.94	80.30	80.00	85.00	78.94	81.33	77.50	70.53	70.00
MINF vs. N	72.50	70.27	72.50	71.72	87.59	87.59	77.50	74.28	72.05	70.00	76.71	70.00
DCM vs. HCM	55.00	52.63	62.50	59.45	59.40	73.81	55.00	50.00	80.71	72.50	60.38	65.00
DCM vs. RV	67.50	64.86	60.00	61.90	72.56	67.54	65.00	65.00	72.78	62.50	51.43	55.00
DCM vs. N	85.00	84.21	85.00	85.00	80.30	60.99	85.00	85.00	73.38	72.50	83.38	80.00
HCM vs. RV	62.50	63.41	70.00	68.42	84.67	74.00	62.50	61.53	78.38	75.00	31.95	50.00
HCM vs. N	75.00	73.68	72.50	73.17	81.25	64.25	65.00	61.11	70.00	67.50	75.76	67.50
RV vs. N	75.00	73.68	72.50	70.27	65.15	55.05	67.50	66.66	67.40	67.50	57.65	55.00
Mean	71.75	70.58	72.50	71.89	75.23	72.34	70.50	68.83	75.83	71.50	64.81	57.95

ACC: Accuracy; MINF: myocardial infarction; DCM: dilated cardiomyopathy; HCM: hypertrophic cardiomyopathy; RV: abnormal right ventricle; N: normal conditions

These findings may be associated with principal components of heart dynamics, such as the rotation movements to describe the left ventricle and particular spatial velocity patterns along the cardiac cycle. Interestingly, each kinematic excelled in discrimination between several conditions. For instance, the vorticity achieved remarkable results in classifying between myocardial infarction versus dilated cardiomyopathy, while the tangential and normal acceleration kinematics had a notable performance in separating dilated cardiomyopathy from control samples.

In a subsequent experiment, we combined the most promising kinematic features as an input block. Table 3 summarizes the results of different kinematic configurations. Considering the correlated nature of such kinematics (there are differential relationships from the optical flow field), no significant enhancement was observed in global accuracy. Nonetheless, there are some remarkable configurations, such as the dilated cardiomyopathy versus normal cardiac condition, that achieve an average accuracy of 92.50% and an F, score of 92.68%, using the coupled configuration of the kinematics: tangential acceleration, divergence, vorticity.

**Table 3.** Obtained accuracy and F_1_ score by using the Automated Cardiac Diagnosis Challenge dataset in the deep learning strategy considering diverse concatenations over time. The kinematic maps herein considered are: a_T_: normal acceleration; a_T_: tangential acceleration; dlv: divergency, and *vor*. vorticity.

Cardiac disease	*(a*_*N*_ *(t),a*_*T*_ *(t), div(t))*		*(a*_*N*_ *(t),a*_*T*_ *(t), vor(t))*		*(a*_*N*_ *(t),div(t), vor(t))*		*(a*_*N*_ *(t),div(t), vor(t))*	
	ACC	F1	ACC	F1	ACC	F1	ACC	F1
MINF vs. DCM	80.00	80.00	77.50	79.06	82.50	82.05	80.00	80.02
MINF vs. HCM	62.50	61.54	62.50	63.41	60.00	57.89	65.00	66.67
MINF vs. RV	85.00	85.00	85.00	85.00	85.00	85.00	80.00	77.76
MINF vs. N	75.00	76.19	85.00	83.34	77.50	75.67	80.00	78.94
DCM vs. HCM	55.00	52.63	50.00	47.36	55.00	52.63	52.50	53.65
DCM vs. RV	70.00	68.42	67.50	66.67	77.50	79.06	67.50	68.29
DCM vs. N	85.00	85.00	90.00	89.47	90.00	89.47	92.50	92.68
HCM vs. RV	67.50	66.67	62.50	65.11	67.50	69.76	55.00	59.09
HCM vs. N	75.00	75.00	87.50	87.80	80.00	78.94	75.00	73.68
RV vs. N	80.00	80.00	85.00	84.21	82.50	82.92	85.00	84.21
Mean	73.50	73.05 75.25		75.14	75.75	75.34	73.25	73.50

ACC: Accuracy; MINF: myocardial infarction; DCM: dilated cardiomyopathy; HCM: hypertrophic cardiomyopathy; RV: abnormal right ventricle; N: normal conditions

### Classification of embeddings from random forest

We considered an additional multi-modal kinematic configuration to enhance the deep representation of each motion feature map. The new cardiac descriptor was evaluated with a random forest classifier. Table 4 shows an experiment using the late fusion of embedding vectors taken from deep representations of the kinematics: normal acceleration, divergence, and vorticity. Following this configuration, the best accuracy result achieved an average of 78.00% and an F, score of 77.55%. Also, it should be highlighted that some experiments achieved a perfect classification score, showing the discrimination capabilities of the three deep kinematic representations.

**Table 4.** Obtained accuracy using the Automated Cardiac Diagnosis Challenge dataset in the binary embedding classification with random forest.

Cardiac disease	*(a*_*n(*_ *t),div(t),vor(t))*			
	Accuracy	F1 score	Precision	Recall
MINF vs. DCM	100.00	100.00	100.00	100.00
MINF vs. HCM	80.00	78.10	85.00	80.00
MINF vs. RV	100.00	100.00	100.00	100.00
MINF vs. N	80.00	81.90	90.00	80.00
DCM vs. HCM	80.00	80.00	86.67	80.00
DCM vs. RV	60.00	66.30	86.67	60.00
DCM vs. N	60.00	60.00	60.00	60.00
HCM vs. RV	60.00	60.00	60.00	60.00
HCM vs. N	80.00	71.11	64.00	80.00
RV vs. N	80.00	78.10	85.00	80.00
Mean	78.00	77.55	81.73	78.00

ACC: Accuracy; a_N_: Normal acceleration; div. divergency; *vor*. vorticity; MINF: myocardial infarction; DCM: dilated cardiomyopathy; HCM: hypertrophic cardiomyopathy; RV:abnormal right ventricle; N: normal conditions

The proposed representations can be implemented as a triage alternative to classify between cine-MRI with any condition or control sequence. We validated the proposed approach in an experiment that merged all labels corresponding to the abnormal cardiac conditions in the same class (myocardial infarction, dilated cardiomyopathy, hypertrophic cardiomyopathy, and abnormal right ventricle). A binary classification was obtained from abnormal conditions versus control sequences, as seen in table 5. The group of embedding vectors - tangential acceleration, divergence, and vorticity kinematics-were employed to perform a late fusion classification. In this last experiment, the proposed approach achieved a remarkable 92.31% accuracy and 91.19% F, score.

**Table 5.** Obtained accuracy using the automated cardiac diagnosis challenge dataset in the multi-class embedding classification with random forest between normal cardiac sequences and any cardiac disease.

Cardiac diseases	Accuracy	F_1_ score	Precision	Recall
Cetin *et al.,* 2017 ^2^	94.00	-	94.00	93.00
Insensee *et al*., 2017 ^9^	92.00	-	92.00	92.00
Khened *et al*., 2017 ^5^	90.00	-	83.40	100.00
Wolterink *et al.,* 2017^10^	86.00	-	84.00	91.00
Ours	92.31	91.19	92.95	92.31

## Discussion

The proposed approach introduced a novel 3D convolutional net to quantify and characterize diverse spatiotemporal motion patterns on the complete cardiac functional cycle. This strategy can recover kinematic maps and obtain a hierarchical deep multi-level representation constructed from a discrimination rule between cardiac conditions. Furthermore, we validated the classification and characterization capabilities of the 3D network operated on the selected cardiac kinematics. The compact embedding outputs were tested and used to train and validate a random forest classifier, achieving remarkable results. The estimation of the deep kinematic representation improved accuracy by over 6% and F, score by 10.88% for each kinematic concerning the original cine MRI sequences. With this proposed kinematic setup, it is possible to combine and enrich a motion representation by convolving simultaneously multiple kinematics for a particular 3D net. This enriched representation (from normal acceleration, divergence, and vorticity) achieved improved performance in discriminating multiple cardiac conditions.

An important feature in this approach is the capability to build compact embedding descriptors that code cardiac conditions and form a topological space to access to an automatic classification. These resultant embedding correspondences may emerge as potential digital biomarkers of cardiac conditions, storing complex correlations achieved from a learning optimization. This approach is promising for implementation in a clinical routine to support triage protocols because of the exhibited performances of around 92.31% accuracy in discriminating between control and any cardiac condition included in this study. Although Cine-MRI is not currently the primary diagnostic study for most cardiac conditions, its growing advantages are becoming increasingly evident, leading to its adoption as a triage scheme for detecting specific cardiac conditions ^17^. Current reports also evidence an effort to introduce such artificial intelligence tools in clinical protocols ^18^. Additionally, the kinematic maps and resultant activations at different layers of hierarchical representation may be relevant during observational analysis.

In the state of the art, much of the methodologies are dedicated to performing ventricle segmentation tasks. Classical indexes such as the ejection fraction and the ventricle volume, among others, can be computed using the resultant volumes ^4^^,^^5^^,^^8^^,^^9^. Indexes are computed from relative differences between end-diastole and end-systole. For instance, Puyol *et al*. proposed a multi-modal atlas that integrates MRI and ultrasound to extract Laplacian motion descriptors, allowing the classification of patients with dilated cardiomyopathy from control subjects ^6^. Additionally, Yang *et al*. proposed a registration strategy to quantify displacement of the left ventricle among temporal consecutive images. However, bypassing abnormal right ventricle analysis, like in that study, might lead to lose information relevant to certain diseases ^7^. Furthermore, Clough *et al*. recovered variational embeddings to discriminate among cardiac diseases ^19^.

Despite the remarkable contributions of these approaches, they remain dependent on proper ventricle segmentation to characterize cardiac pathologies. A main issue of these schemes is the dependency on guided segmentation and the loss of temporal patterns that may be crucial to enrich diagnosis. Likewise, these descriptors are based on known physical features, poorly exploiting potential hidden relationships that may be computed from the (2D +1) information provided by complete cine-MRI sequences.

In contrast, the proposed approach exploits motion relationships that can be calculated from kinematic representation maps but also learned through a 3D representation. This strategy recovers complex motion patterns and may be advantageous to complement typical indices to support expert characterizations of particular cardiac conditions. In this line, some strategies have also captured motion patterns from left ventricles, so their success depends on a proper geometry recovery ^7^^,^^10^.

These experiments evidence a potential use of this strategy as triage support of patients in clinical schemes. Regarding the state-of-the-art, the proposed approach evidenced competitive results regarding accuracy and precision by using several kinematic characteristics and following a leave-one-patient-out cross-validation scheme. This fact showed the robustness of the embedding representation, allowing a reliable classification of cardiac conditions. Furthermore, this approach operates without segmentation requirements, resulting in a more generalized heart representation from cine MRI sequences. The proposed strategy was validated over an open database with real cine MRI sequences over five cardiac conditions. Nonetheless, the capability of the proposed approach should be validated over larger cohorts of data, ideally from different clinical centers. In such a sense, it is expected to report the generalization capacity and the impact of each kinematic map regarding the discrimination capability among cardiac conditions. Also, more middle and end embeddings exploration should be carried out, searching for alternative descriptors of heart observations.

For instance, a topological analysis or a geometrical search over embedding space may be an alternative to validate the discrimination capability. The proposed approach also requires additional processing schemes to include multi-classification from an end-to-end scheme. Future works include studying other types of kinematics that can help to extract relevant patterns, such as attention feature maps. Finally, validation with a larger dataset that includes expert cardiologist annotations and clinical information will be considered to define a possible correlation with medical findings and the advantages and limitations of the approach.

This work proposed a deep volumetric convolutional net to classify cardiac pathologies from MRI sequences. The proposed strategy computes kinematic maps, which allow deep representations to encode complex and hidden kinematics related to the observed pathologies. Two classification schemes were used to validate the proposed approach: 1) An end-to-end scheme and 2) A scheme using embedding descriptors, further mapped into a random forest classifier. The proposed approaches evidence coherent competitive results over an open-access dataset. Future works include the study of geometrical embedding space and validation with larger data cohorts to establish the statistical scope and discriminate among close cardiac pathologies.

## References

[1] World Health Organization (2021). World Health Statistics 2021. Geneva: World Health Organization.

[2] Cetin I, Sanroma G, Petersen SE, Napel S, Camara O, Ballester MA (2019). A radiomics approach to computer-aided diagnosis with cardiac cine-MRI. arXiv.

[3] Chang Y, Jung C (2020). Automatic cardiac MRI segmentation and permutation-invariant pathology classification using deep neural networks and point clouds. Neurocomputing.

[4] Chang Y, Song B, Jung C, Huang L (2018). Automatic segmentation and cardiopathy classification in cardiac MRI images based on deep neural networks. ICASSP.

[5] Khened M, Kollerathu VA, Krishnamurthi G (2019). Fully convolutional multi-scale residual DenseNets for cardiac segmentation and automated cardiac diagnosis using ensemble of classifiers. Med Image Anal.

[6] Puyol-Anton E, Ruijsink B, Gerber B, Amzulescu MS, Langet H, De Craene M (2019). Regional multi-view learning for cardiac motion analysis: Application to identification of dilated cardiomyopathy patients. IEEE Trans Biomed Eng.

[7] Yang D, Wu P, Tan C, Pohl KM, Axel L, Metaxas DN (2017). 3D motion modeling and reconstruction of left ventricle wall in cardiac MRI. Funct Imaging Model Heart.

[8] Isensee F, Jaeger PT, Full PM, Wolf I, Engelhardt S, Maier-Hein KH (2017). Automatic cardiac disease assessment on cine-MRI via time-series segmentation and domain specific features. arXiv.

[9] Wolterink JM, Leiner T, Viergever MA, Isgum I (2017). Automatic segmentation and disease classification using cardiac cine MR images. arXiv.

[10] Qin C, Bai W, Schlemper J, Petersen SE, Piechnik SK, Neubauer S (2019). Joint motion estimation and segmentation from undersampled cardiac MR image. arXiv.

[11] Zhen X, Wang Z, Islam A, Bhaduri M, Chan I, Li S (2016). Multi-scale deep networks and regression forests for direct bi-ventricular volume estimation. Medical Image Analysis.

[12] Punithakumar K, Ben Ayed I, Islam A, Goela A, Ross IG, Chong J (2013). Regional heart motion abnormality detection: An information theoretic approach. Medical Image Analysis.

[13] Zheng Q, Delingette H, Ayache N (2019). Explainable cardiac pathology classification on cine MRI with motion characterization by semi-supervised learning of apparent flow. Medical Image Analysis.

[14] Brox T, Malik J (2011). Large displacement optical flow: Descriptor matching in variational motion etimation. IEEE Trans Pattern Anal Mach Intell.

[15] Varol G, Laptev I, Schmid C (2018). Long-term temporal convolutions for action recognition. IEEE Trans Pattern Anal Mach Intell.

[16] Bernard O, Lalande A, Zotti C, Cervenansky F, Yang X, Heng PA (2018). Deep learning techniques for automatic MRI cardiac multi-structures segmentation and diagnosis: Is the problem solved?. IEEE Trans Med Imaging.

[17] Broncano J, Bhalla S, Caro P, Hidalgo A, Vargas D, Williamson E (2021). Cardiac MRI in patients with acute chest pain. Radiographics.

[18] Fotaki A, Puyol-Antón E, Chiribiri A, Botnar R, Pushparajah K, Prieto C (2022). Artificial intelligence in cardiac MRI: Is clinical adoption forthcoming?. Front Cardiovasc Med.

[19] Clough JR, Oksuz I, Puyol-Antón E, Ruijsink Bram, King AP, Schnabel JA (2019). Global and local interpretability for cardiac MRI classification. Springer.

